# Tamponade dressing versus no dressing after haemorrhoidectomy: multicentre, randomized clinical trial

**DOI:** 10.1093/bjsopen/zrac070

**Published:** 2022-05-27

**Authors:** Mike Ralf Langenbach, Razvan-Valentin Florescu, Andreas Köhler, Jörg Barkus, Jörg-Peter Ritz, Eduart Quemalli, Robert Siegel, Hubert Zirngibl, Rolf Lefering, Lars Bönicke

**Affiliations:** Ev. Krankenhaus Lippstadt, Department of Surgery II, University of Witten/Herdecke, Lippstadt, Germany; Helios St. Elisabeth Klinik Oberhausen, Department of Surgery II, University of Witten/Herdecke, Oberhausen, Germany; Helios Klinikum Duisburg, Duisburg, Germany; Helios Klinikum Niederberg, Velbert, Germany; Helios Klinikum Schwerin, Schwerin, Germany; Helios Klinikum Schwerin, Schwerin, Germany; Helios Klinikum Berlin-Buch, Berlin, Germany; Helios Klinikum Wuppertal, Department of Surgery II, University of Witten/Herdecke, Wuppertal, Germany; Institute for Research in Operative Medicine (IFOM), University of Witten/Herdecke, Cologne, Germany; Helios Klinikum Wuppertal, Department of Surgery II, University of Witten/Herdecke, Wuppertal, Germany

## Abstract

**Background:**

Symptomatic haemorrhoids are a common anorectal disorder. The aim of the study was to investigate whether the omission of tamponade dressings after haemorrhoidectomy reduces postoperative pain without increasing the risk of severe bleeding.

**Method:**

This was an open-label, randomized clinical trial conducted at 14 German hospitals. All patients with third- or fourth-degree haemorrhoids undergoing haemorrhoidectomy were considered eligible for selection in the intervention (no dressing) or control group (tamponade applied). Two co-primary outcomes were analysed by testing hierarchically ordered hypotheses. First, maximum pain intensity within 48 h after surgery was compared between the groups (superiority). This was followed by an analysis of severe bleeding complications, defined as any bleeding requiring surgical re-intervention within 7 days (non-inferiority). Secondary outcomes included health-related quality of life, patient satisfaction, haemoglobin levels, and adverse events.

**Results:**

Out of 950 patients screened, 754 were randomized and 725 received intervention (366 patients in the intervention and 359 patients in the control group). In the group with tamponade dressings, median pain intensity on the 0 to 10 scale was 6 (interquartile range (i.q.r.) 4–7). Patients without tamponade dressings reported significantly less pain (median 5 (i.q.r. 3–7), *P* < 0.001). In each group, five patients (1.4 per cent) experienced severe bleeding. The absolute difference for the severe bleeding rate was −0.03 per cent with the 90 per cent confidence interval ranging from −1.47 per cent to +1.41 per cent, in line with the non-inferiority aim. No significant between-group difference was found for secondary outcomes.

**Conclusions:**

The practice of inserting tamponade dressings after haemorrhoidectomy correlates with increased postoperative pain and does not provide benefits in terms of reduced postoperative bleeding.

**Registration number:**

DRKS00011590

## Teaser

This multicentre, randomized clinical trial investigated the need for tamponade dressings after haemorrhoidectomy

## Introduction

Symptomatic haemorrhoids are a common anorectal disorder^2,3^. Most cases can be sufficiently treated by non-surgical interventions^4–7^. The most used method for treating grade III and IV haemorrhoids is still the Milligan–Morgan haemorrhoidectomy^8–10^, mainly because of its low recurrence rates^8–15^. In Germany, haemorrhoidectomy is mainly an inpatient procedure^15^, as it may be associated with significant postoperative complications such as pain and bleeding. The occurrence of bleeding is often associated with the first passage of hard stool after surgery. Postoperative bleeding can be a serious complication that may range from causing discomfort to being potentially life-threatening. It may occur in 2–6 per cent of cases^16–18^, with early bleeding being more common than late bleeding^8,19,20^. Another postoperative aspect is pain, which can be severe and may delay return to normal activities for several weeks. To prevent post-haemorrhoidectomy bleeding, many surgeons insert an anal tampon, but this practice is rather based on surgical tradition than evidence. Two randomized clinical trials (RCTs) evaluated the effects of different types of tamponade dressings after haemorrhoidectomy: one study compared a simple, non-adherent wound pad with a gelatine sponge plug^21^, whereas others examined whether a calcium alginate dressing is superior to standard gauze packing^22^. Nevertheless, whether a tamponade dressing is necessary after haemorrhoidectomy has never been confirmed in a comparative study. After completing a single-centre trial in 100 patients^23^, a large multicentre trial was designed to evaluate whether the omission of tamponade dressings after haemorrhoidectomy reduces postoperative pain without increasing the risk of severe bleeding. The primary aim of this trial was to compare the maximum pain intensity within 48 h after surgery between the groups (superiority). This was followed by an analysis of severe bleeding complications, defined as any bleeding requiring surgical re-intervention within 7 days (non-inferiority). Secondary outcomes included health-related quality of life, patient satisfaction, haemoglobin levels, and adverse events (AEs).

## Methods

### Design

The NoTamp study was designed as a German national, multicentre RCT^1^. Centre-stratified randomization lists with variable block sizes were prepared by a computer. After consent, participants were randomly allocated in a 1:1 ratio to the tamponade or the no-tamponade group. A centralized web-based tool was used for allocation to treatment groups. Randomization was recommended to be performed at the earliest 12 h before the start of surgery. The patients were not informed about the results of randomization before surgery. The duration of the clinical trial for each randomized patient was 7 days. There was no blinding of participants, physicians, nurses, or outcome assessors. The study received full ethics committee approval of the University of Witten/Herdecke, Germany and was conducted in accordance with Good Clinical Practice. Regular external monitoring visits were carried out at a frequency depending on the number of patients enrolled.

### Setting

The study was conducted in hospital departments with a special focus on colon and rectal surgery with at least one expert in proctology. The study was performed within the Helios Hospital Group. The majority of the participating study sites were hospitals for basic and standard care; three of them were third-level hospitals (maximum care). Uniform quality management and standardized documentation were provided by the Helios Hospital Group.

### Study participants

The target population of this study were patients with symptomatic haemorrhoids, requiring Milligan–Morgan or Parks haemorrhoidectomy. All adult patients (aged 18 years or above) suffered from symptomatic haemorrhoids of grade III or IV. Study participants were fully legally competent and needed to provide written informed consent before randomization. Patients with inflammatory anal diseases such as abscesses, fistulas, or gangrene, and pregnant women were excluded from participation. Patients on anticoagulatory drugs were fully eligible and, in line with the local standard of care, continued their medication before and after surgery.

### Intervention and comparison

In the intervention group, after achieving complete haemostasis no tamponade dressing was placed in the anal canal of the patient. The rectum and anus were cleaned and a pad with high-absorbance capacity was placed on the aperture to absorb potential blood loss. The pad was fixed with mesh pants. In the control group, a tamponade dressing was placed in the patient’s anal canal and lower rectum after complete haemostasis. The intraoperatively inserted tamponade remained at least until first defaecation and possible spontaneous removal, but usually for a maximum of 24 h. Tamponade dressings were used according to clinical routine. The type of tamponade used for the study participants was recorded in case report forms (CRFs). Of note, the study protocol did not interfere with perioperative measures as per local standard of care (such as postoperative use of antithrombotic prophylaxis or Sitz baths). All drugs and other treatments administered were recorded in the CRF.

### Primary and secondary outcomes

Primary outcomes of the NoTamp study were maximum postoperative pain, as measured by a numerical rating scale (NRS) at four time points within 48 h after the surgical procedure. The occurrence of postoperative bleeding with the need for surgical revision within 7 days after haemorrhoidectomy was defined as severe bleeding. Postoperative pain was measured at rest within routine care at 6, 12, 24, and 48 h after the surgical procedure and 7 days after surgery. The most severe pain within 48 h of the surgical procedure was compared between the treatment groups.

In addition, the change in pain intensity from 6 h after the surgical procedure to 12, 24, and 48 h as well as day 7 after the procedure and the development of pain between 24 and 48 h were compared between the intervention and control group. The number of patients in the group without a primary tamponade dressing who required one later were also documented. Further secondary outcomes included the use of analgesics (type and doses), haemoglobin levels (lowest value within 7 days), and AEs (all events regardless of severity or assumed causality).

All expected and unexpected AEs and severe AEs (SAEs) occurring in temporal relation to the clinical trial were documented and compared between the treatment groups. Documentation included the causal relationship with the therapy to be investigated and/or with the previous surgical treatment. For each AE the nature of the event, onset, and end, as well as the severity (mild, moderate, and severe) were documented, particularly with respect to urinary retention, wound infections, fever, and vomiting.

Generic health-related quality of life was quantified with the EuroQoL Group (Rotterdam, The Netherlands) index (EQ-5D™) at screening, on discharge, and on day 7. Each patient’s health state (derived from the EQ-5D™) was assigned an index value in the range 0 to 1 by applying national German preference weights^24^. Patient satisfaction with treatment was measured 7 days after surgery using two validated domains of the Cologne Patient Questionnaire (KPF)^25^. Both subscales covered ‘subjective treatment success’ (three items) and ‘subjective treatment mistakes’ (four items) with all items to be scored on a three-step Likert scale. In addition, a single KPF item on overall patient satisfaction with medical treatment (1 to 5 scale) was analysed.

### Statistics

The sample size was calculated according to the bleeding rate, because this approach was expected to lead to a sample size that also allows a robust conclusion on the other primary outcome. Severe postoperative bleeding was expected to occur in 4 per cent of patients in the control group. In the intervention group, the expected bleeding rate was 8 per cent. Both rates were based on the literature and the pilot study. Assuming that a three-fold increase in the bleeding rate represents a clinically meaningful difference, the non-inferiority limit was defined as 12 per cent. The sample size was thus calculated to exclude a bleeding rate of 12 per cent with 95 per cent certainty (upper limit of a 90 per cent c.i.), based on an expected rate of 8 per cent in the NoTamp group. A total of 866 patients (1:1 ratio) was calculated to be needed for the primary analysis, with a significance level *α* = 0.05 and a power of (1 − *β*) = 0.8 (one-sided test). To allow for about 10 per cent exclusions, the trial was planned with a sample size of 953 patients.

Primary and secondary outcomes were analysed according to a modified intention-to-treat (ITT) principle. This excluded patients who did not undergo haemorrhoidectomy, who did not fulfil all inclusion criteria, or who retracted their consent. The two study hypotheses—superiority with regard to pain, non-inferiority with regard to bleeding—were examined by hierarchically ordered statistical testing. This approach requires an *a priori* specification of the order of hypotheses but allows recycling of the standard level of significance (0.05) for the second hypothesis if the first hypothesis is found to be statically significant^26^. Accordingly, pain intensity was analysed first by applying the non-parametric *U* test (two-sided; *α* = 0.05). In a second step, the rate of severe postoperative bleeding in the intervention group was determined and the 90 per cent confidence interval was calculated. The upper 90 per cent confidence interval margin was compared with the three-fold rate of the bleeding rate observed in the control group. If the 90 per cent confidence interval did not overlap this three-fold rate, non-inferiority of the experimental therapy was assumed (one-sided hypothesis with an error rate of 5 per cent).

All categorical variables were expressed as counts (with percentages) and were analysed with chi-squared or Fisher’s exact test. Continuous data were summarized as mean(s.d.) or median (interquartile range (i.q.r.)) and were compared with the unpaired Student’s *t* test or the Wilcoxon rank sum test, depending on data distribution.

## Results

Between May 2017 and November 2020, 950 patients were screened for eligibility; however, 196 were excluded and a total of 754 patients were enrolled at 14 trial sites (*Table 1*).

**Table 1:** Patient recruitment per study site (*N* = 754)

	Tamponade group (*n* = 373)	No-tamponade group (*n* = 381)
**Helios St. Elisabeth Klinik Oberhausen**	60	61
**Helios Klinikum Wuppertal**	101	102
**Helios Klinikum Berlin-Buch**	16	16
**Helios St. Johannes Klinik**	75	77
**Helios Klinik Lengerich**	3	3
**Helios Klinik Hüls**	9	9
**Helios Klinik Jerichower Land (Burg)**	10	10
**Helios Klinikum Krefeld**	10	12
**DKD Helios Klinik Wiesbaden**	1	1
**Helios St. Josefs-Hospital Bochum-Linden**	9	9
**Helios Kliniken Niederberg**	43	45
**Helios Klinik München Perlach**	0	0
**Helios Klinik Blankenhain**	0	0
**Helios Klinik Wipperfürth**	0	0
**Helios Klinik Siegburg**	2	3
**Helios St. Elisabeth Klinik Hünfeld**	4	4
**Helios Klinikum Schwerin**	30	29

Of note, the COVID19 pandemic led to a steep decline in recruitment and rendered follow-up visits very difficult. Therefore, an unplanned, blinded interim analysis of both primary outcomes was performed. As the rate of severe bleeding was lower than anticipated and recruitment rates remained low, the lead principal investigator decided to stop the clinical trial prematurely at the end of 2020.

**Fig. 1:**
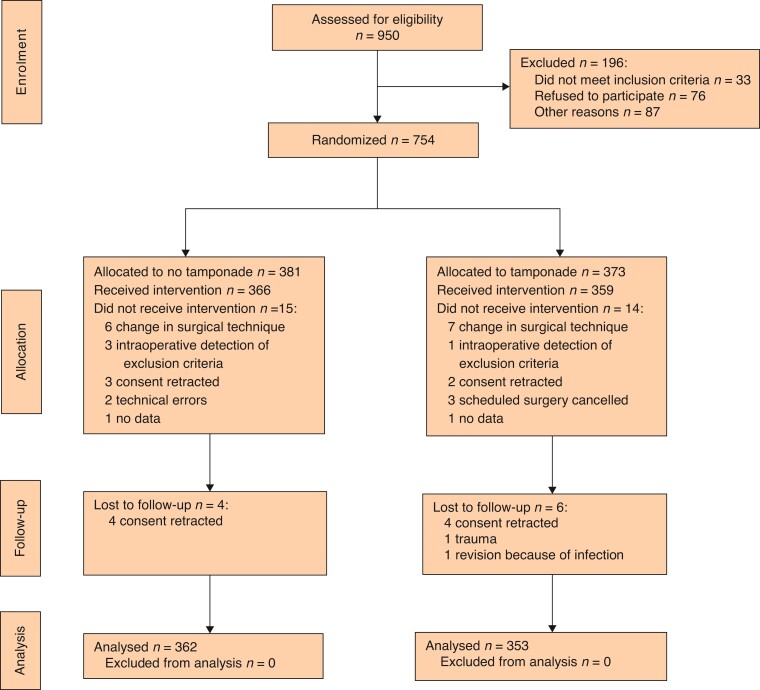
CONSORT diagram template

Therefore, *Fig. 1* displays the trial flow diagram and *Table 2* the baseline characteristics. The ITT population consisted of 725 patients, as in each group 4 per cent of included patients had to be excluded after randomization. Patient characteristics and surgical and anesthesiological interventions were well balanced between the study groups. The patients’ ages ranged from 18 to 89 years and about two-thirds of patients were receiving anticoagulation drugs (mostly heparin).

**Table 2:** Baseline characteristics and operative details

	With tamponade (*n* = 359)	Without tamponade (*n* = 366)
**Age, years, mean(s.d.)**	53 (15)	52 (15)
**Sex ratio (male:female)**	204:155	210:156
**Height, m, mean(s.d.)**	173 (10)	173 (10)
**Weight, kg, mean(s.d.)**	83 (19)	82 (19)
**Anticoagulation medication**		
None	107 (29.9)	109 (29.9)
Aspirin only	16 (4.5)	17 (4.7)
Oral drugs or heparin	235 (65.6)	239 (65.5)
**Grade of haemorrhoids**		
III	189 (52.6)	203 (55.5)
IV	170 (47.4)	163 (44.5)
**Type of haemorrhoidectomy**		
Milligan–Morgan	317 (88.3)	327 (89.3)
Parks	51 (14.2)	53 (14.5)
**Type of anaesthesia** *		
Laryngeal mask	281 (78.3)	279 (76.2)
Intubation	42 (11.7)	44 (12.0)
Spinal	17 (4.7)	27 (7.4)
Local	19 (5.3)	15 (4.1)
Other	22 (6.1)	35 (9.6)
**Additional analgesic intervention**		
Pudendal nerve block	50 (13.9)	47 (12.8)
Local infiltration	9 (2.5)	11 (3.0)

* As more than one type of surgical approach and one mode of anaesthesia could be applied, total numbers add up to slightly more than 100 per cent. Values are *n* (%) unless otherwise indicated.

Among the 359 patients in the control group, 200 (55.6 per cent) received a commercially available tampon made of polyvinyl alcohol (PVA), measuring about 20 mm in diameter and 60–70 mm in length. A gauze packing strip was applied in 101 patients (28.1 per cent). In 40 patients (11.1 per cent), the treating surgeon chose to use a rolled-up gauze swab as tamponade dressing. In about half of these patients, the swab also served to apply adrenaline, lidocaine, or thromboxane to the wound. Finally, 17 patients (4.7 per cent) received an absorbable gelatine sponge to support haemostasis in the anal canal.

### Primary outcomes

**Fig. 2:**
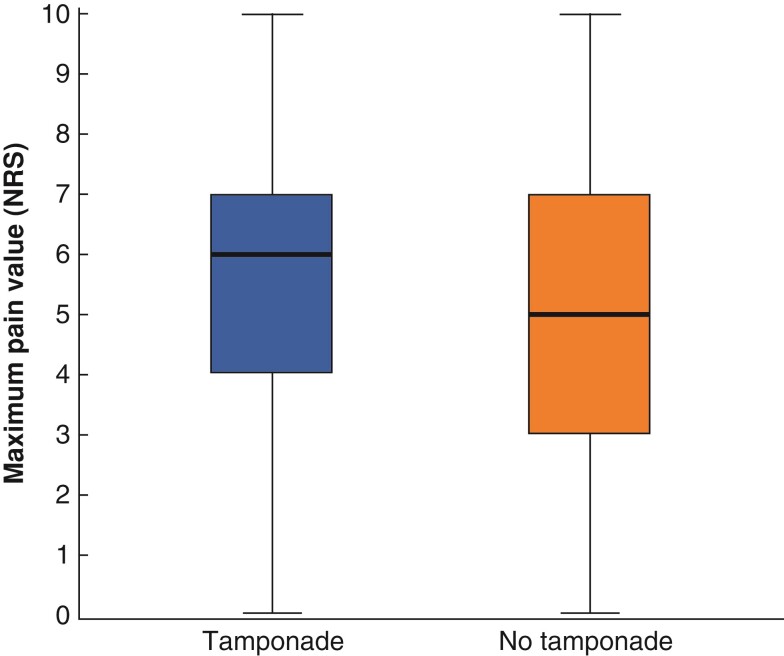
Maximum pain intensity within 7 days after surgery as measured with the numerical rating scale (NRS) *P*<0.001

Maximum pain intensity during the first 48 h after haemorrhoidectomy was higher in patients who had received a tamponade (*P* < 0.001; *Fig. 2*). The median maximum pain intensity was 6 (i.q.r. 4–7) in the control group and 5 (i.q.r. 3–7) in the intervention group. The respective mean(s.d.) values were 5.5(2.4) and 4.8(2.3).

In both groups, the incidence of severe bleeding was low (1.1 per cent). In each group, five bleeding events (in five patients) occurred, of which four were noted before discharge from hospital. The absolute difference for the severe bleeding rate was −0.03 per cent with the 90 per cent confidence interval ranging from −1.47 to +1.41 per cent, in line with the non-inferiority at the conventional *α* level.

### Secondary outcomes

**Fig. 3:**
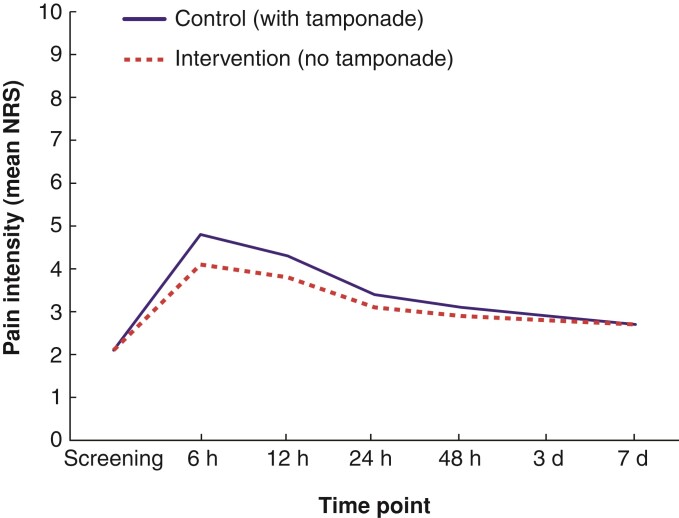
Postoperative pain intensity

**Fig. 4:**
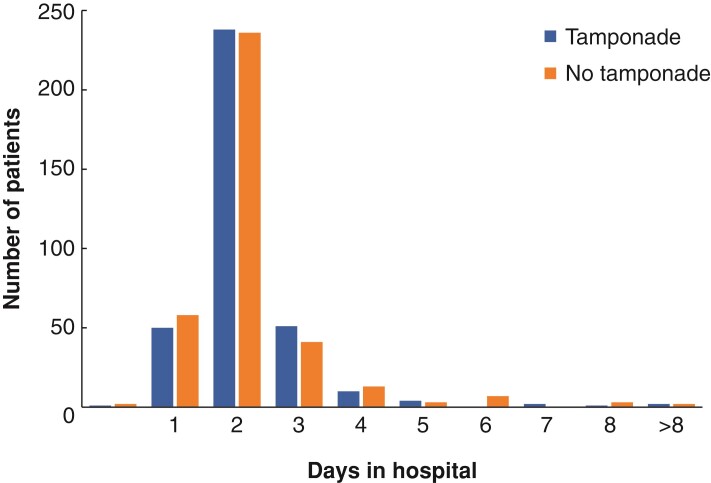
Duration of hospital stay

The course of postoperative pain levels is shown in *Table 3* and *Fig. 3*. Similar to the primary outcome analysis for maximum pain intensity, the between-group comparisons show significantly less pain in the no-tamponade group 6 and 12 h after surgery. The use of analgesics was similar in both groups. Duration of hospital stay was 2 days in more than half of patients, without any difference between groups (*Fig. 4*). Postoperative haemoglobin levels were available for 37 per cent of patients and showed no difference between the intervention group (median 13.4 (i.q.r. 12.2–14.5) mg/dl, *n* = 134) and the control group (13.5 (i.q.r. 12.5–14.9) mg/dl, *n* = 131); *P* = 0.234. Testing of subgroups with different tamponade dressings showed no significant differences in bleeding or pain development.

Postoperative pain levels at 6 h (*P*<0.001) and 12 h (*P*<0.003) were significant lower in control group. NRS, numerical rating scale.

No significant difference was found.

**Table 3:** Pre- and postoperative pain levels, analgesic consumption, duration of hospital stay, and serious adverse events

	With tamponade (*n* = 359)	Without tamponade (*n* = 366)	*P*
Pain intensity, median (i.q.r.)*			
Preoperative	1 (0–4)	1 (0–3)	0.190
6 h after surgery	5 (3–7)	4 (2–6)	0.001
12 h after surgery	4 (2–6)†	4 (2–5)†	0.003
24 h after surgery	3 (2–5)	3 (2–4)	0.165
2 days after surgery	3 (2–4)†	3 (2–4)†	0.152
3 days after surgery	3 (2–4)†	3 (2–4)†	0.279
1 week after surgery	2 (1–4)	2 (1–4)	0.571
**Oral NSAIDs**			
At discharge	251 (69.9)	250 (68.3)	0.688
After 1 week	182 (50.7)	197 (53.8)	0.553
Total number of doses	846	870	NA
**Oral opioids**			
At discharge	90 (25.1)	90 (24.6)	0.932
After 1 week	14 (3.9)	13 (3.6)	0.847
Total number of doses	182	157	NA
**Local analgesics**			
At discharge	135 (37.6)	134 (36.6)	0.818
After 1 week	116 (32.3)	109 (29.8)	0.471
Total number of doses	140	148	NA
**Duration of hospital stay, days, median (i.q.r.)**	2 (2–2)	2 (2–2)	NA
**SAEs**	7	3	0.562

i.q.r., interquartile range; NSAIDs, non-steroidal anti-inflammatory drugs; SAE, serious adverse event; NA, not available. *Pain intensity was recorded with the 0 to 10 numerical rating scale. †Completeness of pain level was less than 95 per cent for the time points at 12 h (89 per cent), 2 days (62 per cent), and 3 days (52 per cent), thus requiring replacement by last observation carried forward. Values are *n* (%) unless otherwise indicated.

Ten serious AEs (three *versus* seven in intervention *versus* control group) were recorded in eight patients: urinary retention, wound infections, fever, and vomiting (*Table 3*). Prolonged duration of hospital stay or readmission was required for surgical revision (six patients), treatment of bleeding (two patients), or urinary retention (two patients). A total of 57 patients experienced a total of 65 AEs (serious or non-serious). Both the number of AEs (30 *versus* 35) and the number of patients affected (29 *versus* 28) did not differ between the groups. Health-related quality of life as measured with the EQ-5D™ was similarly high in both groups (*Table 4*). The same applied to patient satisfaction, although patients without a tamponade dressing were slightly more satisfied with medical treatment (*Tables 4 and*  *5*).

**Table 4:** Health-related quality of life and patient satisfaction

	With tamponade (*n* = 359)	Without tamponade (*n* = 366)	*P*
**EQ-5D™, median (i.q.r.)** *			
Preoperative	92.6 (79.5–92.6)	90.3 (77.0–92.6)	0.444
At discharge	90.3 (75.1–92.6)	90.3 (79.5–92.6)	0.492
1 week after surgery	92.6 (79.5–92.6)	91.4 (79.5–92.6)	0.822
**Overall patient satisfaction with medical treatment** **†**			0.083
Dissatisfied	4 (1.2)	0 (0)	
Slightly dissatisfied	1 (0.3)	1 (0.3)	
Neither dissatisfied, nor satisfied	14 (4.4)	6 (1.8)	
Slightly satisfied	45 (14.0)	42 (12.9)	
Satisfied	257 (80.1)	277 (85.0)	

*Maximum value is 92.6. †The wording of the Cologne Patient Questionnaire item was: ‘How satisfied were you with medical treatment?’ Data are missing for 38 (10.6 per cent) and 40 (10.9 per cent) patients respectively. i.q.r., interquartile range; EQ-5D™, EuroQoL Group index. Values are *n* (%) unless otherwise indicated.

**Table 5:** Patient satisfaction as measured by the Cologne Patient Questionnaire on day 7

	With tamponade (*n* = 359)	Without tamponade (*n* = 366)
**Subjective treatment success** *		
‘I believe that the therapy performed had an effect.’	Full agreement 57.1% (205 of 311)	Full agreement 58.2% (213 of 324)
‘Due to therapy, I feel better.’	Full agreement 52.4% (188 of 313)	Full agreement 53.8% (197 of 317)
‘The treatment has improved my quality of life.’	Full agreement 49.0% (176 of 303)	Full agreement 50.3% (184 of 316)
**Subjective treatment mistakes** *		
‘There were complications in my treatment’	Full disagreement 75.5% (271 of 319)	Full disagreement 74.0% (271 of 325)
‘During my treatment, there were medical problems.’	Full disagreement 78.6% (282 of 326)	Full disagreement 76.5% (280 of 327)
‘I had the impression that the medical staff made mistakes in my treatment’	Full disagreement 81.6% (293 of 325)	Full disagreement 81.7% (299 of 330)
‘I believe that the wrong treatment option was chosen.’	Full disagreement 82.5% (296 of 326)	Full disagreement 83.9% (307 of 329)

*All items were scored as ‘fully disagree’, ‘slightly disagree’, ‘slightly agree’, and ‘fully agree’. Data are presented as percentages (with absolute numbers) only for the largest response category.

## Discussion

The results of the present trial confirm the findings of the pilot investigation^23^. First, it was shown that the use of tamponade dressings correlates with increased postoperative pain after haemorrhoidectomy. This result was statistically significant already in the pilot trial, but that study was too small to rule out a relevant increase in bleeding complications when omitting tamponade dressings. With the results herein reported, it can be safely postulated that the omission of tamponade dressings after haemorrhoidectomy does not correlate with an increased risk of bleeding. As the total incidence of AEs was also slightly lower in the no-tamponade group, it is unlikely that this result was affected by the restriction of the primary outcome to only severe bleeding events. Furthermore, it can be ruled out that severe bleeding events were misclassified as non-serious (or went completely unrecorded), because the trial was centrally monitored. Finally, haemoglobin levels did not differ between groups. In the literature, there are indications that the occurrence of bleeding after haemorrhoidectomy is a rare event (0.9–10 per cent), but the type of surgical procedure can represent an independent risk factor^27,28^. One study found out that the removal of symptomatic second to fourth-degree haemorrhoids with the LigaSure procedure causes significantly more bleeding after surgery than surgery after Ferguson (11.9 per cent *versus* 5.1 per cent; *P* = 0.015)^27^. The LigaSure vessel sealing system was developed to reduce anal spasm and pain after haemorrhoidectomy. In their retrospective study, the authors describe the LigaSure procedure and constipation as independent risk factors for bleeding after haemorrhoidectomy. Anal canal packing was not routinely performed, except when homeostasis was doubtful. With the LigaSure system a number of short-term benefits were described, such as reduced intraoperative blood loss, operating time, and postoperative pain.

Compared with the findings of the pilot trial, where pain intensity was reduced from 6 to 4 points, pain reduction in the present trial was smaller (from 6 to 5 points). As both trials had no blinding, the placebo effect must be considered, although in this case, the application of the tamponade dressing in the control group is more likely to result in a possible nocebo effect. In addition, effect sizes of about 1 or 1.5 points on a 10-point pain scale do not exceed the minimal clinically important difference (MCID). Several studies on acute pain estimated the MCID to lie in the range between 1 and 1.5 points^29,30^. After haemorrhoidectomy, an average patient will therefore not consider their pain worse if pain intensity is 1 point higher due to a tamponade dressing. On the other hand, even a minor burden seems unwarranted if this disadvantage is not counterbalanced by some advantage of a tamponade dressing.

The necessity of external pressure dressings is currently being studied by a research group in China^31^. In a pilot study, they found that pressure dressings increased the risk of urinary retention to more than 40 per cent. Urinary retention was a surprisingly rare complication in the present trial, which may be because external pressure dressings are not the standard of care in Europe.

This trial has a few limitations: first, the types of tamponade dressings varied between study centres. Although this variability of study interventions may have biased relevant differences, the aim of the trial was to answer a broad, practical question, even if such an approach does not allow specific insights into the mechanisms of local haemostasis, perianal inflammation, pain, or wound healing.

The premature closure of the trial might be seen as a threat to the study’s validity and statistical power; however, the statistical power of the study would have been very similar, even if recruitment had been completed. With regard to internal validity, it should be noted that data-driven stopping of clinical trials increases the risk of bias, but in the present case, halting the trial was entirely due to organizational and financial reasons in the context of the COVID19 pandemic.

Ideally, the present trial results and their implementation should be accompanied by quality assurance approaches. Systematic surveillance of post-surgical complications, including severe haemorrhage, and routine recording of pain levels are potentially appropriate measures; registries are a useful adjunct to RCTs, as they can be used first to detect very rare complications, and second, to determine whether benefits seen in RCTs extend to clinical practice in all settings.

## Supplementary Material

zrac070_Supplementary_data

## Data Availability

Data will be made available at the website of the NoTamp study. Anonymized raw data are available upon request from the corresponding author or the trial statistician.
